# Speech Fluency Improvement in Developmental Stuttering Using Non-invasive Brain Stimulation: Insights From Available Evidence

**DOI:** 10.3389/fnhum.2021.662016

**Published:** 2021-08-11

**Authors:** Pierpaolo Busan, Beatrice Moret, Fabio Masina, Giovanni Del Ben, Gianluca Campana

**Affiliations:** ^1^IRCCS Ospedale San Camillo, Venice, Italy; ^2^Department of Life Sciences, University of Trieste, Trieste, Italy; ^3^Department of General Psychology, University of Padua, Padua, Italy

**Keywords:** developmental stuttering, neuromodulation, transcranial electrical stimulation, transcranial magnetic stimulation, motor networks

## Abstract

Developmental stuttering (DS) is a disturbance of the normal rhythm of speech that may be interpreted as very debilitating in the most affected cases. Interventions for DS are historically based on the behavioral modifications of speech patterns (e.g., through speech therapy), which are useful to regain a better speech fluency. However, a great variability in intervention outcomes is normally observed, and no definitive evidence is currently available to resolve stuttering, especially in the case of its persistence in adulthood. In the last few decades, DS has been increasingly considered as a functional disturbance, affecting the correct programming of complex motor sequences such as speech. Compatibly, understanding of the neurophysiological bases of DS has dramatically improved, thanks to neuroimaging, and techniques able to interact with neural tissue functioning [e.g., non-invasive brain stimulation (NIBS)]. In this context, the dysfunctional activity of the cortico-basal-thalamo-cortical networks, as well as the defective patterns of connectivity, seems to play a key role, especially in sensorimotor networks. As a consequence, a direct action on the functionality of “defective” or “impaired” brain circuits may help people who stutter to manage dysfluencies in a better way. This may also “potentiate” available interventions, thus favoring more stable outcomes of speech fluency. Attempts aiming at modulating (and improving) brain functioning of people who stutter, realized by using NIBS, are quickly increasing. Here, we will review these recent advancements being applied to the treatment of DS. Insights will be useful not only to assess whether the speech fluency of people who stutter may be ameliorated by acting directly on brain functioning but also will provide further suggestions about the complex and dynamic pathophysiology of DS, where causal effects and “adaptive''/‘‘maladaptive” compensation mechanisms may be strongly overlapped. In conclusion, this review focuses future research toward more specific, targeted, and effective interventions for DS, based on neuromodulation of brain functioning.

## Introduction

Developmental stuttering (DS) is a neurodevelopmental disorder characterized by speech dysfluencies such as blocks and repetitions, especially occurring during the first part of words and sentences. Associated motor symptoms (such as muscular spasms) may be evident, especially in the orofacial districts, which could not be strictly related to the motor programs of the intended utterances of speakers. DS appears during childhood (Yairi and Ambrose, 2005), normally between 3 and 9 years of age, when the brain is rapidly developing and increasing skills related to complex motor tasks, such as speech. It affects about 5% of the pediatric population (e.g., Andrews and Harris, 1964), but the majority of DS in children is able to recover a normal speech fluency in a “spontaneous” unassisted way. In the rest of DS, it persists in adulthood (about 1% of the total adult population) (e.g., Drayna et al., 1999). In the most severe cases, DS significantly affects the quality of life of people who stutter. At present, DS is considered as a “multifactorial” disorder characterized by genetic and neural abnormalities (e.g., Alm, 2004; Drayna and Kang, 2011; Craig-McQuaide et al., 2014; Barnes et al., 2016; Etchell et al., 2018; Benito-Aragon et al., 2020; Busan, 2020; Chang and Guenther, 2020). In this context, even if various ways of intervention may be proposed, a “crucial” solution is still not available (e.g., Brignell et al., 2020; Connery et al., 2020; see also Qureshi et al., 2021). Considering that stuttering may be viewed as a “motor timing disorder” (e.g., Etchell et al., 2014; see also Chang et al., 2016), dysfluencies are usually improved when people who stutter are facing with external “cues” (such as choral speech, the use of a metronome, and altered auditory feedback) (e.g., Foundas et al., 2004; Etchell et al., 2014; Park and Logan, 2015) inducing a change in the spontaneous rhythm of speech. However, these solutions are usually not easy to be used in an “ecological” environment. As a consequence, current strategies are mainly based on behavioral “fluency-shaping” interventions, which are aimed at improving the motor/speech skills of persons who stutter (e.g., the Lidcombe or the Camperdown Program) (e.g., Onslow et al., 1997; O'Brian et al., 2003). This may be obtained by modifications, such as slowing speech rhythm and execution, resulting in the management of dysfluencies in a more favorable way. In addition, considering that these techniques may affect speech naturalness, they may be sometimes difficult to be generalized, applied, and effectively maintained. Psychotherapy or cognitive-behavioral interventions may be also adopted, especially when facing with “secondary” (but still important) effects of stuttering, such as anxiety, social phobia, and the avoidance of speech situations (see Maguire et al., 2020). The combination of all these approaches may typically result in the management of DS in a better way.

Similarly, pharmacotherapy also shows promising results (e.g., Maguire et al., 2020). However, also in this case, a definitive solution is still not available. Different active ingredients are demonstrated to be useful in improving dysfluencies and associated motor symptoms of DS, especially in the case of influencing dopaminergic activity (e.g., Maguire et al., 2000a,b, 2004, 2010, 2019; Busan et al., 2009; Tavano et al., 2011). Research is now concentrated on finding the solution of possibly balancing the positive outcomes with good tolerability (e.g., Maguire et al., 2019, 2020).

Finally, sporadic reports also describe the single cases of DS that are resulted as incidentally improved in case of using invasive interventions (such as deep brain stimulation) to treat other conditions and of targeting the thalamus or the basal ganglia system (e.g., Maguire et al., 2012; Thiriez et al., 2013).

As a consequence, new ways of treatment should be always needed and considered to improve (or alleviate) stuttering in a better way. In this perspective, very recent evidence has suggested to combine neuromodulation techniques with more “conventional” interventions. Neuromodulation is able to “boost” brain functioning by means of non-invasive brain stimulation (NIBS) techniques (see updated guidelines, Antal et al., 2017; Lefaucheur et al., 2017; Rossi et al., 2021). Interestingly, NIBS has been recently and increasingly proposed to investigate its potential in modulating the brain functioning of people who stutter, with an aim to improve their speech fluency. Here, most recent advancements in treating stuttering with NIBS will be reviewed to combine this evidence with the previously available information about the neural dynamics of DS. This will provide additional insights into the brain functioning of persons who stutter, as well as further suggestions for evidence-based treatments leveraging on NIBS.

## The Defective Neural Circuits of DS

Previous neuroimaging research identified a wide pattern of abnormalities characterizing the neural structures of people who stutter (e.g., Brown et al., 2005; Craig-McQuaide et al., 2014; Neef et al., 2015; Etchell et al., 2018, for comprehensive reviews and meta-analyses). These abnormalities have a role in various tasks, such as motor planning, preparation, and execution, especially in case of their involvement in complex sequences, such as speech (e.g., Alm, 2004; Etchell et al., 2018; Chang and Guenther, 2020; see also, for neural modeling, Civier et al., 2010, 2013). Furthermore, they may interact with cognitive, attention, or emotional brain networks (e.g., Craig-McQuaide et al., 2014; Chang et al., 2018).

As a consequence, a series of “neural markers,” typical of DS, may be extrapolated (e.g., Brown et al., 2005; Neef et al., 2015). They can be summarized as (1) *the hypoactivation of speech and the motor structures of the left hemisphere* (e.g., Watkins et al., 2008; Chang et al., 2009; Desai et al., 2017; compare with Neef et al., 2015); (2) *a larger hyperactivation of the homologous regions of the right hemisphere* (e.g., Brown et al., 2005; Chang et al., 2009; compare with Neef et al., 2015); (3) *the impaired/abnormal structure of a cortical gray matter and white matter*, thus resulting in altered connectivity patterns, which are responsible for an unsuccessful neural communication (e.g., Sommer et al., 2002; Beal et al., 2007; Watkins et al., 2008; Chang et al., 2011); (4) an *altered neural activity in cortico-basal-thalamo-cortical circuits* (e.g., Wu et al., 1995; Giraud et al., 2008; Watkins et al., 2008; Chang and Guenther, 2020), also in relation to a “defective” dopaminergic regulation (e.g., Wu et al., 1997; Alm, 2004; compare with Alm, 2021; Turk et al., 2021); and (5) *altered sensorimotor interactions* in a neural level (e.g., audio–motor interactions, “sensory-to-motor” feedback/transformation, or “motor-to-sensory” projections) (e.g., Beal et al., 2010, 2011; Cai et al., 2012, 2014a; Daliri and Max, 2015; Saltuklaroglu et al., 2017; Jenson et al., 2020), which may easily result in the alterations of the functional communication among the brain regions (e.g., Busan et al., 2019).

Interestingly, these abnormalities are not only strictly related to speech tasks but can also be evident during non-speech motor tasks (e.g., Sommer et al., 2003; Chang et al., 2009; Neef et al., 2011; Busan et al., 2013, 2016) or even at rest (e.g., Wu et al., 1997; Sommer et al., 2003; Alm et al., 2013; Busan et al., 2013; Desai et al., 2017; Chang et al., 2018). The altered networks highlighted in DS are fundamental to the programming/execution of complex motor sequences, especially when they are voluntary or internally driven, as is the case for cortico-basal-thalamo-cortical networks (see Busan, 2020; Chang and Guenther, 2020). Hypoactivations or hyperactivations of speech/motor neural networks usually involve various brain regions, for example, the inferior frontal cortex, sensorimotor (primary and associative) regions, the temporal cortex, and subcortical structures, thus constituting complex and reciprocally connected circuits that have been shown to have a role in speech/motor planning and articulation. For example, neuroimaging and direct electrical stimulation data suggest the fundamental role of inferior frontal regions in mediating a series of representations, ranging from speech-related sensory information (e.g., in case of collaboration with the temporal cortex) to corresponding motor and articulatory codes that are made available to the motor regions (e.g., Deletis et al., 2014; Rogić et al., 2014; Flinker et al., 2015; see Etchell et al., 2018, for a recent and comprehensive review in DS). The lower activity of these structures may be more easily evident in the left hemisphere, whereas highly activated networks may be more easily, but not exclusively, evident in the right one (e.g., Wu et al., 1995; Brown et al., 2005; Watkins et al., 2008; Chang et al., 2009; Neef et al., 2015; Desai et al., 2017; Connally et al., 2018). The patterns of impaired/abnormal activity may be responsible for DS but may also reflect the continuous attempts that the brain of people who stutter is running, trying to avoid dysfluencies (thus resulting in an “adaptive” or a “maladaptive” compensation) (e.g., Giraud et al., 2008).

Another feature of DS is the presence of abnormalities in a cortical gray matter (e.g., Foundas et al., 2001, 2003, 2004; Beal et al., 2007; Chang et al., 2008), and white matter (e.g., Sommer et al., 2002; Jäncke et al., 2004; Watkins et al., 2008; Connally et al., 2014; Neef et al., 2018). In this context, for example, abnormalities may be evident in case of considering the indexes of the hemispheric lateralization of frontotemporal structures (e.g., lower asymmetry in the planum temporale or in the prefrontal regions of people who stutter) (e.g., Foundas et al., 2001, 2003, 2004). Similarly, DS may result in the reduction of a cortical gray matter in brain regions such as associative motor cortices, as well as in deeper cortical regions such as the anterior cingulate cortex (e.g., Chang et al., 2008; Garnett et al., 2018). Impaired structural connectivity among the brain regions may be also evident in a compatible manner, thus influencing sensorimotor integration and motor/speech implementation. Also in this case, the networks of the left inferior frontal cortex and sensorimotor regions are usually the most affected circuits (e.g., Sommer et al., 2002; Chang et al., 2008; Watkins et al., 2008; Connally et al., 2014) while the increase in homologous right hemispheric ones may be the result (e.g., Jäncke et al., 2004). However, alterations have been highlighted also in broader structures, such as long-range fascicles (e.g., longitudinal and/or arcuate fascicles connecting anterior and posterior parts of the brain) (e.g., Cykowski et al., 2010; Connally et al., 2014; Chow and Chang, 2017; Neef et al., 2018), the corpus callosum (mainly connecting the homologous regions of the two hemispheres) (e.g., Cykowski et al., 2010; Civier et al., 2015; Chow and Chang, 2017), or corticospinal and corticobulbar tracts (that allow to drive motor commands toward muscular effectors) (e.g., Watkins et al., 2008; Connally et al., 2014). A particular mention should be made for the frontal aslant tract (FAT) that connects the supplementary motor area (SMA) and the inferior frontal cortex (Catani et al., 2012). FAT permits the exchange of motor information between these regions, with a role in speech production, planning, sequencing, and initiation (e.g., Vassal et al., 2014; Fujii et al., 2015; Kinoshita et al., 2015; Chernoff et al., 2019; Dick et al., 2019). More specifically, while the left FAT seems to be more involved in the planning (and coordination) of sequential motor acts (such as speech), the right homologous region should be more involved in an “inhibitory” motor control as well as in the resolution of conflicts among “competitive” actions (see, for example, Kinoshita et al., 2015; Dick et al., 2019; for a perspective in stuttering compare with Neef et al., 2018; La Corte et al., 2021). Interestingly, the FAT has been shown to be defective in DS (e.g., Kronfeld-Duenias et al., 2016; Neef et al., 2018), but has been also reported to be increased in the right hemisphere of people who stutter (e.g., Misaghi et al., 2018). In this context, Kemerdere et al. (2016) reported that the direct electrical stimulation of FAT, during neurosurgery, induced stuttering-like dysfluencies in fluent speakers. Similar evidence was reported by Corrivetti et al. (2019), showing that the stimulation of FAT, fronto-striatal tract, corpus callosum, and corticospinal tract (also involving cortical regions such as the precentral gyrus and the pars opercularis) may result in speech motor disturbances such as speech arrest, stuttering-like dysfluencies, and vocalizations. Similarly, Kinoshita et al. (2015) reported that the FAT and the fronto-striatal tract (mainly connecting the SMA “complex” and the basal ganglia) may cooperate to coordinate the motor aspects of self-initiated actions and speech: in fact, patients experienced intraoperative inhibition of movements and speech during the direct electrical stimulation of these tracts.

Developmental stuttering is also strongly related to the altered activation of the cortico-basal-thalamo-cortical system, in which subcortical structures (i.e., basal ganglia) are considered as fundamental hubs (e.g., Alm, 2004; Giraud et al., 2008; Watkins et al., 2008; Toyomura et al., 2011, 2015; Craig-McQuaide et al., 2014; Etchell et al., 2018; Chang and Guenther, 2020). Similarly, their cortical targets, such as the SMA, may play a central role (e.g., Busan, 2020). The SMA “complex” may be divided into a “proper” SMA region and a pre-SMA: the former is massively connected with the motor cortex, whereas the latter is strongly related with executive/cognitive regions (e.g., prefrontal or temporoparietal cortices) as well (e.g., Picard and Strick, 1996; Johansen-Berg et al., 2004; Klein et al., 2007; Nachev et al., 2008; Ruan et al., 2018). The SMA is fundamental to the correct implementation of complex (internally driven) motor sequences (as well as in motor inhibition), thanks to the information shared with regions such as the basal ganglia, prefrontal regions, and the inferior frontal cortex (Ikeda et al., 1999; Seitz et al., 2006; Narayana et al., 2012; Rochas et al., 2013; Ruan et al., 2018). In this context, the altered functioning of the basal ganglia frequently highlighted in DS (e.g., Wu et al., 1995, 1997; Alm, 2004; Watkins et al., 2008) and often associated with stuttering severity (e.g., Giraud et al., 2008; Toyomura et al., 2011) is likely to result in a “disequilibrium” among excitatory and inhibitory motor signals (compare with Busan et al., 2016, 2017). This may affect the correct functioning of connected cortical targets, thus resulting in the defective programming/implementation of complex motor sequences. Similarly, a direct stimulation (or injuries) of the SMA “complex” and related networks may result in induced stuttering or speech dysfluencies (e.g., Alexander et al., 1987; Abe et al., 1992, 1993; Ackermann et al., 1996; Van Borsel et al., 1998, 2003; Dinoto et al., 2018; see also Penfield and Welch, 1951; Ackermann and Riecker, 2011).

Evidence also suggests that the abnormal functioning of the basal ganglia in people who stutter may be due to an imbalance of dopaminergic activity (Wu et al., 1997; see also Alm, 2021; Turk et al., 2021, for a recent perspective): pharmacological interventions with antidopaminergic drugs may be useful, in a compatible manner, to “restore” a near-to-normal neural activity in DS, especially in the basal ganglia and Broca's region (Maguire et al., 2021).

Finally, DS seems to be characterized by the presence of altered sensorimotor interactions. This may be evident in case of considering the audio–motor interactions that have been suggested to be impaired in people who stutter (e.g., Beal et al., 2010, 2011; Cai et al., 2012, 2014a; Daliri and Max, 2015). However, impaired sensorimotor interactions may be evident also at a more global level, i.e., in case of considering the brain rhythms that are useful for motor implementation and/or sensorial gating (e.g., Saltuklaroglu et al., 2017; Jenson et al., 2020). These weaknesses may easily result in altered functional interactions in the brain circuits of persons who stutter, especially in case of considering demanding tasks such as effective (and timely) speech programming and implementation (e.g., Chang et al., 2019). These “functional” disruptions may result in “poor” neural synchronization (or “delayed” neural activity) among the networks that are useful for motor programming and execution (e.g., Salmelin et al., 2000; Etchell et al., 2014; Busan et al., 2019).

Thus, it is evident that DS is a very complex and dynamic motor disorder likely to be more “general” than the one that is previously hypothesized (e.g., Ludlow and Loucks, 2003; see also Smits-Bandstra et al., 2006; Smits-Bandstra and De Nil, 2007), involving broader brain regions and neural networks (e.g., Chang et al., 2018, 2019; Busan et al., 2019; see also Etchell et al., 2018, for a comprehensive review). Neural activity related to the causal aspects of the disturbance and also to compensation attempts may be overlapping and very difficult to discriminate, resulting in the more complicated understanding of DS neural processes. For example, the modulatory effects of emotional processes on motor programs, in DS (e.g., Yang et al., 2017; Toyomura et al., 2018), should be further investigated and better discriminated (see Craig-McQuaide et al., 2014). Despite the complex scenarios that are suggested to explain DS, researchers agree on two aspects so far: (1) the left hemisphere and cortico-striato-thalamo-cortical impairments can be causally related to stuttering and (2) the right hemispheric (over)activity can be more easily interpreted as compensatory and related to the life-long attempts of overcoming dysfluencies (e.g., Chang et al., 2008) (please also consider that an excessive inhibitory activity of the right hemisphere—perhaps related to “maladaptive” attempts—has been suggested to have a role in maintaining—or worsening—stuttering; see Neef et al., 2018).

These observations may be translated into useful suggestions to improve the interventions for people who stutter: the inferior frontal cortex, motor cortices (e.g., the SMA “complex”), and temporoparietal cortex are often a part of altered neural circuits related to stuttering (Etchell et al., 2018). In this light, they could be the target of non-invasive interventions, which aimed at restoring the impaired/abnormal functioning of DS neural networks, thus hypothetically resulting in an improved speech fluency. In this perspective, NIBS may be a promising opportunity and also a potential “game-changer,” which aimed at improving the currently available treatments of stuttering.

## NIBS Methods and Neuromodulation

Non-invasive brain stimulation allows to directly interact with the functioning of a neural tissue. Therefore, it has been used to obtain further and “real-time” information about the impaired/abnormal motor processes that are the peculiarities of DS (see Busan et al., 2017 for a recent review). Overall, NIBS modulates the activity of the brain networks to modify their functioning (e.g., Miniussi and Ruzzoli, 2013; Miniussi et al., 2013). In both transcranial magnetic stimulation (TMS) and transcranial electrical stimulation (tES), the repeated administration of an externally applied (non-invasive) stimulation may promote neural plasticity, possibly resulting in long-term potentiation (LTP) or long-term depression (LTD) of the neural targets (e.g., Miniussi and Ruzzoli, 2013). This may be evident immediately after the stimulation session, lasting for a discrete amount of time afterwards (e.g., Pirulli et al., 2013; Fertonani et al., 2014; Moret et al., 2019). In general, NIBS is stated to act with the addition of “noise” to the neural system (Miniussi et al., 2013). Stimulation effects (e.g., facilitation or inhibition) are possible as a result of interactions with experimental tasks and also with the actual state-dependency of the brain (Silvanto and Pascual-Leone, 2008; Miniussi et al., 2013). As a consequence, stimulation may be coupled with rehabilitation techniques (e.g., physiotherapy or behavioral interventions) to further promote plasticity and possibly result in better outcomes (e.g., Pirulli et al., 2013; Moret et al., 2018). TMS can induce the activation of the stimulated neural tissue, thanks to the delivery of a magnetic field, using a dedicated coil (eight-shaped or double-cone coils allow to obtain the “focal” stimulations of the neural target): neural structures that are perpendicular to the induced magnetic field will be stimulated. TMS may be used to investigate the functionality of the motor system as well as the role of sensory/associative/cognitive brain regions at rest and in a wide range of tasks (Walsh and Alvaro Pascual-Leone, 2003). In case of inducing long-lasting effects in the neural system, TMS is applied by delivering the repeated pulses, for a certain period of time, on the targeted brain region: a “high-frequency” stimulation (e.g., >5 Hz) (see Maeda and Pascual-Leone, 2003) usually results in LTP-like effects, increasing the excitability of the stimulated networks (e.g., Miniussi and Ruzzoli, 2013). On the other hand, a “low-frequency” stimulation (e.g., 1 Hz) (see Maeda and Pascual-Leone, 2003) usually results in LTD-like effects, lowering the excitability of the stimulated networks (e.g., Miniussi and Ruzzoli, 2013).

Similarly, tES has been developed to modulate the activity of the targeted neural regions and networks. It uses low amounts of current to modulate the activity of the stimulated brain tissue, thus increasing or lowering its excitability depending on the stimulation protocol (e.g., Paulus, 2011a; Fertonani and Miniussi, 2017; Reed and Cohen Kadosh, 2018). The most commonly used protocols are (1) *transcranial direct current stimulation (tDCS)*, where an anode or a cathode is placed on the scalp (in correspondence of the region of interest) modulating the resting membrane potential of neurons, generally resulting in increased or decreased excitability of the neural target, respectively (a reference electrode of opposing voltage is also applied in a different cephalic or extracephalic position) (see Nitsche et al., 2008; Nitsche and Paulus, 2011); (2) a *transcranial alternating current stimulation (tACS)* is delivered using the patterns of sinusoidal current at a defined frequency (e.g., 20 Hz); this interacts with the physiological oscillations of the brain, possibly resulting in their better entrainment/synchronization, thus modulating (possibly improving) their functioning (e.g., Battleday et al., 2014); and (3) a *transcranial random noise stimulation (tRNS)*, delivering an alternating current with random amplitudes and frequencies. In this case, stimulation may be delivered at high random frequencies (e.g., 100–640 Hz; thus being able to increase cortical excitability; see Moret et al., 2019), or at lower random frequencies (e.g., 0–100 Hz; this may result in opposite excitability effects—as compared to a high-frequency stimulation—or in non-significant modulations of the stimulated cortex) (see Terney et al., 2008; Campana et al., 2016); tRNS may rely on the stochastic resonance phenomenon (e.g., Moss et al., 2004; McDonnell and Ward, 2011; Miniussi and Ruzzoli, 2013; Pavan et al., 2019), theoretically enhancing the “sensitivity” of the stimulated tissue (see Miniussi and Ruzzoli, 2013).

However, research on the NIBS field is constantly resulting in new possibilities of brain stimulation. For example, in case of considering the theta burst stimulation (TBS), Huang et al. (2005) uses bursts of pulses of a high-frequency repetitive TMS (rTMS) (e.g., 50 Hz), re-proposed at a “theta-rhythm” (e.g., every 200 ms) (Huang et al., 2005). This allows to reduce the total duration of the stimulation, inducing faster LTP-like or LTD-like phenomena depending on the characteristics and protocols of the stimulation (e.g., Huang et al., 2005). Similarly, new tES protocols have been proposed, resulting in “combined” stimulation protocols: for example, the transcranial pulsed current stimulation (tPCS) “optimizes” stimulation outcomes to combine tonic and phasic effects (Jaberzadeh et al., 2014). Anyway, progress in this field is running: in this sense, for example, the techniques of transcranial pulse stimulation with ultrasounds (Beisteiner et al., 2019), transcranial pulsed magnetic field stimulation (Rodger et al., 2012), or transcranial static magnetic field stimulation (Oliviero et al., 2011) are also in development, thus likely to result in new possibilities and protocols, also for patients, in the near future.

Non-invasive brain stimulation is normally characterized by a limited spatial resolution in a neural level. This problem may be partially mitigated by using advanced neuronavigation methods (based on the magnetic resonance information) as well as by using more “focal” TMS coils and tES configurations. In the latter case, for example, the use of particular montages and electrodes [e.g., high definition-tES (HD-tES)] may be useful to reduce unspecific stimulations, thus limiting the heterogeneity of findings and effects (e.g., Edwards et al., 2013; Masina et al., 2021). Fortunately, NIBS methods are usually well-tolerated when safety guidelines are followed in terms of admitted protocols and populations (e.g., Antal et al., 2017; Lefaucheur et al., 2017; Rossi et al., 2021).

Non-invasive brain stimulation techniques are currently and extensively employed in the functional improvement of various motor/cognitive functions in healthy participants (e.g., Moret et al., 2019; Masina et al., 2021) as well as in experimental rehabilitation trials (see Hamilton et al., 2011; Campana et al., 2014; Moret et al., 2018). However, while protocols may be sometimes ineffective in the healthy population (e.g., Wiltshire and Watkins, 2020), the involvement of clinical (or subclinical) participants may result in an effective advantage for their conditions. In this context, a wide range of neural impairments may be considered, such as stroke (e.g., aphasia) (see Hamilton et al., 2011; Marangolo et al., 2013a,b; Khedr et al., 2014), neurodegenerative diseases (e.g., Alzheimer's disease and Parkinson's disease) (e.g., Cotelli et al., 2011; Goodwill et al., 2017), and psychiatric disorders (e.g., schizophrenia, depression, obsessive-compulsive disorder, attention deficit, hyperactivity disorder, etc.) (e.g., Maeda and Pascual-Leone, 2003; Hasan et al., 2016; Palm et al., 2016). For example, previous evidence of acquired motor/language disorders reported additional improvements when tES and behavioral therapies are combined (see Hamilton et al., 2011). As a consequence, considering that, in DS, the effect of conventional (e.g., behavioral) techniques is usually limited, the additional modulatory effect induced by NIBS should be investigated. This should be done to assess whether (1) additional improvements in speech fluency (i.e., the efficacy of interventions) and (2) better brain functioning of people who stutter (also increased understanding of the complex neural dynamics of DS) might be obtained.

## NIBS to Improve Speech Fluency in DS

The use of NIBS, in DS, takes place very recently. A PubMed search (last check: June 2021) using the keywords “transcranial,” “stimulation,” and “stuttering” resulted in a total of 31 articles, but only 6 were the studies using a neuromodulatory approach in DS. Three additional reports taken into consideration in this review (one in a “pre-print” form) were found thanks to a more general search on the web. The first attempt, which is aimed to verify the modulatory effects of tES on the brain functioning of people who stutter, has been published in 2017 (Chesters et al., 2017). Previously, Garnett and den Ouden (2015) implemented a trial of the single sessions of anodal/cathodal tDCS in a group of 11 participants with DS (compared to a group of 20 fluent speakers), stimulating the posterior part of the left superior temporal gyrus (2 mA for 20 min; no findings—i.e., improved or impaired speech—approached significance). However, the aim of this study was the implementation of more effective sham methods for HD-tES. Furthermore, a report using the peripheral transcutaneous electrical nerve stimulation (applied on the jaw and on the neck) to treat the participants with persistent stuttering and concomitant orofacial disorders (e.g., bruxism) also exists (Merlo, 2020). This is a multiple case study conducted to allow a successive better application of more conventional (i.e., behavioral) fluency-shaping techniques. The results showed a reduction in stuttering frequency and severity after the intervention, suggesting that the peripheral stimulation of facial muscular districts may have positive effects on some DS participants. In this case, it should be considered that peripheral structures are always in interaction with the brain systems (especially at a sensorimotor level) (see Schuhfried et al., 2012), thus possibly modulating their functioning [in this context, see also De Bonis et al. (2020), for a case study of the disappearance of persistent DS after an iatrogenic lesion of the facial nerve].

However, at present, neuromodulatory interventions in DS are mainly addressed to two different (but related) neural targets: inferior frontal regions (which include the Broca's area) and the SMA “complex.” These cortical areas are considered as a part of complex and wider speech/motor networks, comprising different structures such as the temporoparietal cortex, associative and primary sensorimotor regions, and the basal ganglia. In addition, inferior frontal regions and the SMA “complex” are directly interconnected through axonal fibers constituting distinct fascicles, such as the FAT, which have been shown to have a role in DS (e.g., Kronfeld-Duenias et al., 2016; Misaghi et al., 2018; Neef et al., 2018). In the following sections, available evidence will be presented by considering the anatomical targets of stimulation. Finally, a brief perspective on current ongoing trials will be also offered.

## NIBS to Improve Speech Fluency in DS: The Stimulation of the Inferior Frontal Cortex

Chesters et al. (2017) investigated the effects of a single session of tDCS on the indexes of speech fluency in 16 adults who stutter. They used anodal or sham tDCS at 1 mA for 20 min. In the sham condition, the stimulation ramped up and down within the first 45 s of the protocol. The anode electrode was placed over the left inferior frontal cortex, in correspondence of the FC5 electrode position (according to the common systems of EEG electrode placement), whereas the cathode was placed over the right supraorbital ridge. The size of electrodes used for stimulation was 5 cm × 7 cm. tDCS was associated to a behavioral training, in which participants had to read in a “choral speech” mode following a recorded voice. Speech fluency (primary outcome: the percentage of stuttered syllables; secondary outcomes: stuttered syllables per minute, speaking rate) was assessed before the stimulation session, immediately after and 1 h later: indexes were obtained from sentence reading, passage reading, and spontaneous conversation. The findings suggested a general effect of choral speech practice irrespective of real or sham stimulations, especially in the sentence reading task. However, although these findings did not show a significant difference between real and sham tDCS, a trend was found to suggest an improvement of speech fluency in real tDCS while measuring reading and aftereffects (i.e., 1 h later) related to conversational tasks. Thus, even if no significant tDCS-induced improvements in speech fluency have been individuated in this single-session study (likely to be influenced by heterogeneity in stuttering severity and variations across evaluations, as suggested by the authors), the increased excitability of the left inferior frontal region (and its effect on related networks) should be further considered to evaluate the outcomes on the speech fluency of people who stutter.

In a successive study, the same research group conducted a randomized, double-blind, controlled trial, in which the same protocol was proposed for people who stutter in the 5 consecutive days of treatment (Chesters et al., 2018). In addition, real tDCS (15 adult participants) was compared to sham stimulation (15 adult participants). Neuromodulation was associated to a behavioral intervention (i.e., choral speech and metronome-timed speech). Speech fluency was evaluated before and during treatment, as well as 1 and 6 weeks after the end of the tDCS sessions: the main outcome was the evaluation of dysfluencies (the percentage of stuttered syllables) during reading and conversation in addition to the scores obtained from the stuttering severity instrument-4 (SSI-4) (Riley, 2009), and a subjective evaluation of the psychosocial impact of stuttering [Overall Assessment of the Speaker's Experience of Stuttering (OASES)] (Yaruss and Quesal, 2006). The findings suggest that speech fluency was generally improved in case of using real tDCS, in particular in case of its evaluation 1 week after the conclusion of the stimulation sessions. Interestingly, improvements were also maintained 6 weeks later, especially in the case of considering dysfluencies in the reading task. In conclusion, the left inferior frontal cortex is a possible neural target, in which neuromodulation may have “positive” effects, in stuttering.

In this context, Yada et al. (2019) investigated the effect of single sessions of tDCS on the speech fluency of adults who stutter, during a reading task, stimulating various neural targets in both the hemispheres. More specifically, they investigated the effect of tDCS on putative Broca's and Wernicke's regions in the left hemisphere as well as on their homologs placed in the right one. Anodal and cathodal stimulations were administered in two different sessions (each session composed of a total of 13 adult participants). Stimulation comprises the four blocks of real tDCS (2 mA; the total duration of the stimulation 210 s per block; the size of the electrodes: 5 cm × 7 cm). Active electrodes were placed on the following locations based on the EEG scalp positions: between F7 and FC5 (i.e., putative Broca's region in the left hemisphere), between TP7 and C5 (i.e., putative Wernicke's region in the left hemisphere), between F8 and FC6 (i.e., putative homolog of the Broca's region in the right hemisphere), and between TP8 and C6 (i.e., a putative homolog of the Wernicke's region in the right hemisphere). The return electrode (either cathodal or anodal, according to the planned session) was always placed on the supraorbital region, which is contralateral to the stimulation site. Sham stimulation was also proposed for participants by using one of the already described montages (in this case, the stimulation site was rotated among the four target sites and participants) and delivering the current only for the initial 30 s of the session at 1 mA. During stimulation sessions, participants were asked to read a passage aloud, thus evaluating the effect of the different protocols on the indexes of speech fluency (the percentage of stuttered “moras,” i.e., the Japanese phonological units—comparable to syllables—were considered). The findings mainly suggest that anodal and cathodal sessions of the same brain regions resulted in “opposite” patterns of evidence. More specifically, the most evident result suggested that cathodal stimulation was able to induce a significant improvement of speech dysfluencies (registered in the reading task) in case of its delivery in the frontal regions of the right hemisphere. A “qualitative” (i.e., non-significant) improvement was also observed in case of using anodal stimulation on the contralateral homologous brain regions. This evidence is compatible with the suggestion that the speech/motor networks of both hemispheres may be “causally” and differently involved in determining dysfluencies (e.g., Neef et al., 2016, 2018). Indeed, while the left hemisphere activity is usually impaired in DS and may need to be “boosted,” the activity of the right hemisphere is classically considered as related to compensatory reactions to stuttering. In this context, the right frontotemporal networks may play an “adaptive” compensatory role in fluency enhancements (e.g., Alm, 2004; Etchell et al., 2014; Neef et al., 2015, 2018; see also Giraud et al., 2008; Busan et al., 2019). On the other hand, “maladaptive” reactions to dysfluencies may be also present. As a consequence, the hyperactivity of right frontal/prefrontal networks (also involved in proactive and reactive actions and motor inhibition) (see Neef et al., 2018) may sometimes speculatively result in the worsening of DS symptoms (e.g., Neef et al., 2016, 2018).

In case of considering TMS, Le Guilloux and Compper (2018) described the case of an adult with a severe and persistent stuttering, who received high-frequency rTMS on the left inferior frontal cortex in combination with a speech therapy. TMS was delivered at 10 Hz (30 trains of 5 s, with an inter-train interval of 30 s; 1,500 pulses per session), using the intensity of 80% of the resting motor threshold (RMT) of a hand muscle. A total of 10 sessions were proposed for the participant (5 days per week) every 3 months (three cycles were reported). The left inferior frontal cortex was identified using neuronavigation, and the figure-of-eight TMS coil was oriented with a handle in an anterior-to-posterior and a medial-to-lateral direction. The authors report a progressive improvement of speech fluency, resulting in a “quasi-normal” speech in the end of the third cycle of stimulation.

Finally, Tezel-Bayraktaroglu et al. (2020) started from the evidence to demonstrate an overactivation of the homologous speech-related regions of the right hemisphere in DS. As a consequence, they used an inhibitory rTMS protocol (1 Hz; 800 monophasic pulses at 90% of the RMT of a hand muscle) on different subregions of the right inferior frontal gyrus (8 adult male participants who stutter). In particular, using a figure-of-eight coil, they stimulated the portions of the pars opercularis [Brodmann area (BA) 44], the anterior and the posterior pars triangularis (BA45; please note that BA44 and BA45 are commonly reported to compose the Broca's region, in the left hemisphere), and the portions of the mouth primary motor cortex (BA4), individuated by means of a neuronavigation system. A single TMS session stimulating a specific target region was administered on different days. The coil was normally maintained at 45° tangentially to the scalp and with the handle pointing back. Real stimulation was compared to sham stimulation. Stuttering severity was evaluated before and after stimulation sessions (calculating the percentages of stuttered syllables), recording reading and conversational samples. Interestingly, in case of the stimulation of the anterior pars triangularis (BA45), opposite effects were seen: conversational samples resulted in the worsening of dysfluencies while the evaluation of reading samples resulted in an improvement of stuttering. The authors suggest that these two tasks may be differently detailed in the brain of people who stutter: the “burden” on the speech networks, during a “spontaneous” conversation, can lead to an increased involvement of the right hemisphere, especially in case of evidence of impairments on the left one (as is the case in DS). Thus, the enhanced activity of the right hemisphere may have a compensatory effect, especially in case of considering the tasks that require an augmented demand of linguistic and internally driven motor processes [i.e., conversation; compare with recent perspectives advanced in Alm, 2021], likely increasing the probability that dysfluencies may appear. As a consequence, in this case, the reduction of cortical excitability might have worsened stuttering. On the other hand, simpler, more “automatic,” or “repeated” tasks (such as reading) may result in a decreased stuttering (e.g., Sandak and Fiez, 2000; Ambrose, 2004). Therefore, these tasks could have benefits in case of decreasing the activity of the specific frontal/prefrontal neural circuits of the right hemisphere, perhaps related to attention/control processes and motor inhibition (compare with Neef et al., 2016, 2018), thus speculatively allowing to increase the involvement of “opposite” (or homologous, in the case of inferior frontal cortex) left hemispheric brain regions (see Neumann et al., 2003). Similarly, these tasks may require the involvement of further and different (e.g., frontotemporal) bilateral networks, in DS, which may be theoretically needed for the better management of “rhythmic” or “external” (i.e., sensorial) cues, such as those arising in case of reading (see, for a comprehensive perspective in stuttering, Etchell et al., 2014; compare with Alm, 2004; Neef et al., 2015; Chang et al., 2016).

**Figure 1:**
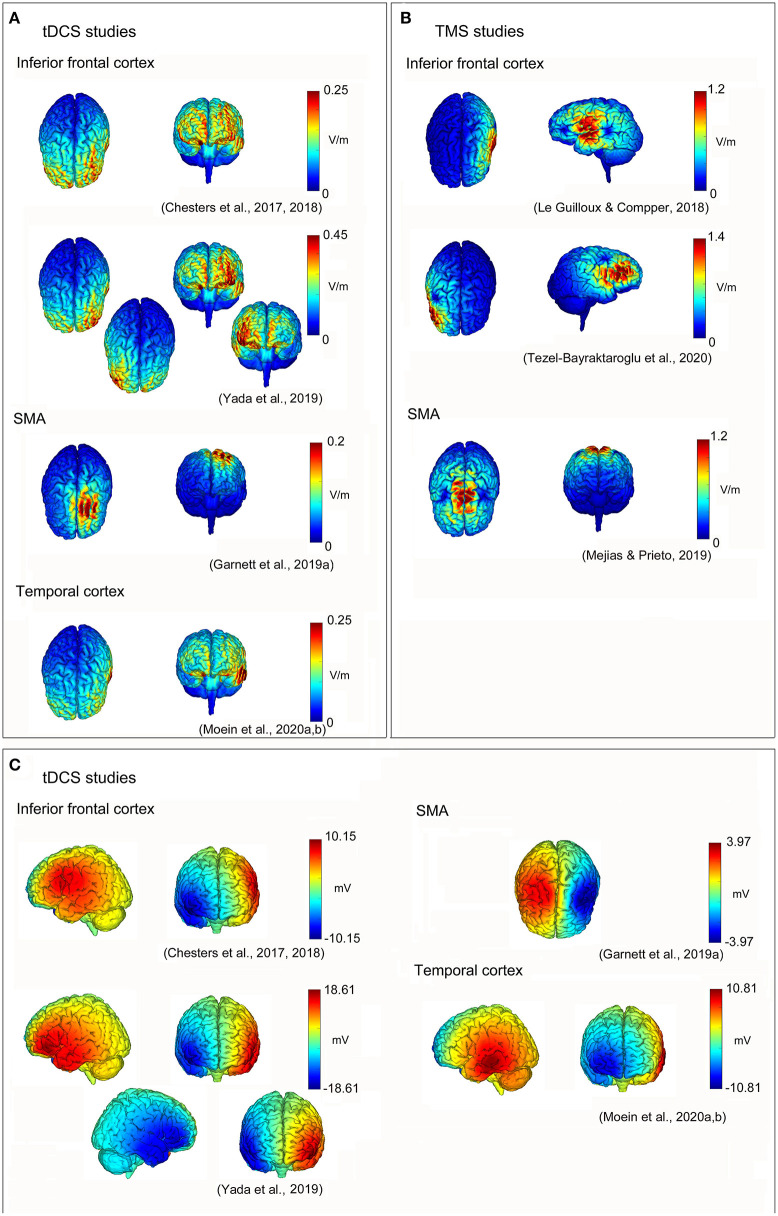
Non-invasive brain stimulation (NIBS) neuromodulation in developmental stuttering (DS) to improve speech fluency. Reconstructions of electric field strengths for every reviewed study: **(A)** absolute values of the electric fields of transcranial direct current stimulation (tDCS) studies estimated using “SimNIBS” (Thielscher et al., 2015; https://simnibs.github.io/simnibs/build/html/index.html); **(B)** absolute values of the electric fields of transcranial magnetic stimulation (TMS) studies estimated using “SimNIBS” (Thielscher et al., 2015; https://simnibs.github.io/simnibs/build/html/index.html); and **(C)** positive (red) and negative (blue) values of the electric fields of tDCS studies estimated using “NIC2” (Neuroelectrics, Barcelona, Spain).

In conclusion, the neuromodulation of the inferior frontal cortex may be useful to improve some aspects of speech fluency in DS, also in follow-up evaluations. This could be obtained by increasing the activity of the inferior frontal cortex (and related networks) in the left hemisphere, and/or inhibiting the activity of homologous regions (and networks) in the right one (see Figure 1) [the induced electrical fields of the reviewed studies have been estimated using the free toolbox “SimNIBS”; (Thielscher et al., 2015); https://simnibs.github.io/simnibs/build/html/index.html-, and the software “NIC2”—Neuroelectrics, Spain—based on the reported stimulation parameters; also, a summary of parameters and of the findings of the reviewed studies is reported in Table 1]. A better understanding of the combined interactions between the left and right hemisphere motor/speech regions will be helpful to obtain further improvements in speech fluency and brain functioning in DS.

**Table 1:** Summary of characteristics and findings of the reviewed studies using non-invasive brain stimulation (NIBS) neuromodulation in developmental stuttering (DS) to improve speech fluency.

					**Parameters entered to perform the e-fields simulations**	
**Study (alphabetical order)**	**Principal type of NIBS applied (and stimulation parameters)**	**Characteristics of experimental participants and experimental tasks**	**Principal cortical target**	**Main finding**	**Reconstructed electrode/coil features and orientations used for electrical fields calculations (Figure 1)**	**Estimated electrode/coil positions on the scalp (SimNIBS coordinates x,y,z)used for electrical fields calculations (Figure 1)**
Chesters et al. (2017)	Anodal tDCS (1 mA, 20 min.; electrode/sponge size 5 × 7 cm)	16 right-handed adult males who stutter; Single session (sham controlled), plus behav. training	Left inferior frontal cortex (anode on FC5; cathode on the right supraorbital ridge)	No significant difference between conditions in indexes of speech fluency (qualitative effect of real tDCS)	Electrode/sponge (anode: short side horizontal; cathode: long side horizontal); electrode thickness 1 mm; sponge thickness 5 mm	Anode: pos −73.53, 15.33, 54.01; dir −73.53, 25.33, 54.01; Cathode: pos 43.88, 77.36, 36.94; dir 50.88, 87.72, 36.94
Chesters et al. (2018)	Anodal tDCS (1 mA, 20 min.; electrode/sponge size 5 × 7 cm)	30 adult males who stutter (15 on tDCS, 15 on sham); 5 daily consecutive sessions (sham controlled), plus behav. Training	Left inferior frontal cortex (anode on FC5; cathode on the right supraorbital ridge)	Improvement in indexes of speech fluency at the end of real tDCS, and in follow-up	Electrode/sponge (anode: short side horizontal; cathode: long side horizontal); electrode thickness 1 mm; sponge thickness 5 mm	Anode: pos −73.53, 15.33, 54.01; dir −73.53, 25.33, 54.01; Cathode: pos 43.88, 77.36, 36.94; dir 50.88, 87.72, 36.94
Garnett et al. (2019a)	Anodal HD-tDCS (1.5 mA, 20 min.; ring electrodes)	14 adults who stutter (3 females); Single session (sham controlled), plus behav. training	Left SMA (anode on FCz; cathode on FC1)	Attenuation of correlations between stuttering severity and right thalamo-cortical activity by real tDCS	Sintered Ag/AgCl ring electrodes (outer radius 12 mm; inner radius 6 mm; total height of the ring 13 mm); electrode thickness 1 mm	Anode: pos −0.64, 22.92, 110.10; dir −0.64, 32.92, 110.10; Cathode: pos −34.38, 21.98, 102.08; dir −34.38, 31.98, 102.08
Le Guilloux and Compper (2018)	High-Freq. rTMS (10 Hz; 30 trains of 5 sec.; inter-train interval 30 sec.; intensity: 80% RMT)	1 right handed adult male who stutter; 10 daily consecutive sessions every 3 months (X 3), plus behav. training	Left inferior frontal cortex (pars operculo-orbicularis)	Improvement in indexes of speech fluency at the end of the first treatment, and in follow-up	Figure-of-eight coil, diameter of every wing: 70 mm; handle oriented in an anterior-to posterior, and medial-to-lateral direction	Coil: pos −79.88, 3.6, 40.63; dir −79.07, 3.58, 46.72
Mejías and Prieto (2019)	High-Freq. rTMS (10 Hz; 60 trains of 5 sec.; inter-train interval 25 s; intensity: 120% RMT)	1 right handed adult male who stutter; 15 daily consecutive sessions, plus behav. training	Bilateral SMA (MNI -x,y,z- 0, 6, 66)	Improvement in indexes of speech fluency after 5 sessions, maintained in follow-up	Figure-of-eight coil, diameter of every wing: 70 mm; handle pointing backwards, parallel to the scalp midline	Coil: pos −1.98, 1.58, 89.44; dir 0.39, 25.65, 109.49
Moein et al. (2020)^1^	Anodal tDCS (1 mA, 20 min.; electrode/sponge size 5 × 7 cm)	50 right handed adults who stutter (two groups); 6 daily consecutive sessions (sham controlled), plus behav. training and DAF	Left superior temporal gyrus (anode on T3; cathode on Fp2)	Improvement in indexes of speech fluency at the end of real tDCS, and in follow-up *(preliminary findings)*	Electrode/sponge (anode: long side horizontal; cathode: long side horizontal); electrode thickness 1 mm; Sponge thickness 5 mm	Anode: pos −80.96, −16.17, 20.47; dir −75.12, −15.75, 6.13Cathode: pos 29.18, 86.77, 34.05; dir 29.0, 85.17, 41.5
Tezel-Bayraktaroglu et al. (2020)	Low-Freq. rTMS (1 Hz; 800 pulses, 90% RMT)	8 right-handed adult males who stutter; Single sessions (sham controlled)	Right inferior frontal cortex—anterior pars triangularis	Improvement in indexes of speech fluency for reading tasks; worsening in indexes of speech fluency for conversational tasks	Figure-of-eight coil, diameter of every wing: 75 mm; coil tangentially oriented at 45 degrees, with the handle pointing back	Coil: pos 40.65, 23.79, 37.23; dir 66.5, 39.61, 50.46
Yada et al. (2019)	Anodal/Cathodal tDCS (2 mA, 210 sec.; electrode/sponge size 5 × 7 cm)	13 right-handed adults who stutter (4 females); Single sessions (sham controlled), plus behav. training	Left/Right inferior frontal cortex (anode/cathode between F7 and FC5, and between F8 and FC6, respectively; cathode/anode on the right/left supra-orbital region, respectively—return electrodes)	Improvement in indexes of speech fluency for the reading task, after real tDCS	Electrode/sponge (anode: long side horizontal; cathode: long side horizontal); electrode thickness 1 mm; sponge thickness 5 mm	Anode (F7 and FC5): pos −73.16, 27.77, 41.35; dir −74.49, 30.68, 28.26Cathode (F8 and FC6): pos 73.16, 27.77, 41.35; dir 74.49, 30.68, 28.26Anode (left supraorbital ridge; return electrode): pos −29.18, 86.77, 34.05; dir −29.0, 85.17, 41.5Cathode (right supraorbital ridge; return electrode): pos 29.18, 86.77, 34.05; dir 29.0, 85.17, 41.5

## NIBS to Improve Speech Fluency in DS: The Stimulation of the SMA “Complex”

The SMA “complex” is another promising candidate for the efficient neuromodulation of the impaired/abnormal networks in stuttering (see Busan et al., 2019; Busan, 2020). For example, Garnett et al. (2019a) used a single-session tDCS (compared to sham) in the left supplementary motor regions of people who stutter. They recruited 14 adult DS participants (3 women), and a HD-tDCS protocol was administered with an aim to improve stimulation focus. More specifically, anodal HD-tDCS was delivered to stimulate at 1.5 mA for 20 min, placing the anodal electrode on the FCz EEG scalp position, whereas the cathodal electrode was placed on the FC1 EEG scalp position. In sham stimulation, the current was ramped up and down over the first 30 s of the session, repeating this procedure at the end of the stimulation. During the protocol, participants were asked to read aloud while following the rhythm of a metronome. Effects on speech dysfluencies and brain activity were investigated: the indexes of stuttering severity (the main outcome was calculated as the percentage of stuttering-like dysfluencies) and functional MRI (during aloud choral reading and solo reading) were recorded before and after stimulation sessions. Speech fluency improved in both real stimulation and sham sessions, especially in reading samples (qualitatively, higher improvements were noticeable after the real tDCS session). No differences were evident in brain activity, except the presence of an association between the stuttering severity (i.e., SSI-4 scores) and the right thalamo-cortical activity that “disappeared,” after stimulation, in the real tDCS group. Authors suggest that these findings may be related to previous evidence suggesting that stuttering severity is positively correlated with connection strengths in regions such as the right anterior thalamic radiation and the right frontal cortex (by means of the FAT fascicle) (Neef et al., 2018). Compatibly, previous works showed the existence of correlations between the stuttering severity and the activity of deeper structures (e.g., the basal ganglia): interestingly, these correlations were evident before a behavioral therapy intervention (i.e., “fluency-shaping”), but not after it (e.g., Giraud et al., 2008; compare with Neumann et al., 2003).

Based on the previous works (i.e., Neef et al., 2018; Busan et al., 2019), Mejías and Prieto (2019) investigated the feasibility of using rTMS to improve speech fluency in DS. More specifically, evidence indicating the presence of “delayed” neural networks in stuttering related to an inefficient activation of the SMA “complex” (Busan et al., 2019) and evidence indicating the presence of an increase in structural connectivity of the motor response/inhibition networks (including the SMA “complex”) (Neef et al., 2018) in people who stutter were considered to implement a single-case study. An excitatory, high-frequency protocol (10 Hz; stimulation intensity: 120% of the RMT; 3,000 pulses applied per session, delivering 60 trains of 5 s with 25 s of inter-train interval) was delivered to the SMA “complex,” bilaterally, on 15 consecutive working days (i.e., 3 weeks). TMS was applied through a figure-of-eight coil, and the SMA “complex” was identified by means of neuronavigation. In the inter-train intervals, the participant was instructed to read aloud following the pacing of a metronome. The authors evaluated rTMS effects calculating the percentages of the stuttered syllables in tasks such as spontaneous conversation, as well as recording SSI-4 scores. This was done before the treatment and after 5, 10, and 15 sessions. The findings suggest a fast and strong decrease in speech dysfluencies (i.e., after five sessions) that was maintained at the end of the treatment.

These studies suggest that also the SMA “complex” may be an effective neural target to improve speech fluency and brain functioning in DS. Indeed, the evidence is still poor and should need to be expanded. However, the evidence of a neural effect allowing to “dissolve” a life-long relation between the discrete patterns of neural activity and stuttering severity after only one session (Garnett et al., 2019a) as well as the evidence of a “boosted” speech fluency effect after a longer treatment (Mejías and Prieto, 2019) suggests that the therapeutic neuromodulation of the SMA “complex” in DS deserves further investigation and consideration (see Figure 1 and Table 1).

## NIBS to Improve Speech Fluency in DS: Current and “In-Progress” Trials

Based on the reviewed studies, further investigation of the neuromodulatory effects of NIBS in DS should be encouraged. In this context, starting from the evidence that altered neural activity of the auditory cortex is evident in stuttering (e.g., Daliri and Max, 2015), the report registered by Moein et al. (2020) considers the effects of combining tDCS (stimulating primary and secondary auditory brain regions) and delayed auditory feedback (DAF) training. DAF is a well-known approach, allowing to temporarily enhance speech fluency in DS (e.g., Foundas et al., 2004). This is a randomized, double-blind, sham-controlled study involving a total of 50 participants and 6 stimulation sessions: all participants receive DAF (60 ms delay during oral reading, monolog, and conversation); the experimental group also receives anodal tDCS on the left superior temporal gyrus (1 mA for 20 min; anodal electrode placed in correspondence of the T3 EEG position; cathodal electrode placed on the right prefrontal region, on Fp2 EEG position; the surface of electrodes 5 cm × 7 cm; see Figure 1), whereas the control group receives sham stimulation. The primary outcome measurement is the percentage of stuttered syllables, whereas the secondary outcomes are the scores obtained from SSI-4 (Riley, 2009) and OASES (Yaruss and Quesal, 2006) scales. Indexes are obtained from reading, monolog, and conversation tasks, recording before and after treatments as well as in follow-up evaluations that are foreseen 1 and 6 weeks after the end of the interventions. A reduction in the percentage of stuttered syllables is hypothesized in the group who undergoes real tDCS and DAF, as well as improvements in physical concomitants (as individuated by SSI-4) and the quality of life. Compatibly, preliminary results (Moein et al., under review)^1^ suggest that stuttering is significantly reduced immediately 1 and 6 weeks after the real tDCS and DAF intervention (compared to the control group).

Similarly, other controlled clinical trials evaluating the effect of neuromodulation in DS are currently running as reported on the web (e.g., https://clinicaltrials.gov/ct2/show/NCT03437512; https://clinicaltrials.gov/ct2/show/NCT03335722). In these studies, anodal tDCS (1–2 mA for 20 min; 5 days of stimulations associated with speech training) is applied on brain regions such as the left frontotemporal cortex. In the end of treatments (and in follow-up phases), improvements in the indexes of speech fluency and brain functioning (e.g., the neurophysiological indexes of motor/speech/auditory networks related to DS) will be evaluated.

## Neuromodulatory NIBS in DS: Insights From Available Evidence And Neural Modeling of Stuttering

Available findings suggest that neuromodulatory NIBS may be a promising approach in improving speech fluency and brain functioning in DS. Data are still limited and need to be expanded: in fact, sometimes only “qualitative” evidence has been reported. The weakness of this evidence may depend upon several aspects. The first one may have to do with the heterogeneity of measures used to evaluate speech fluency. In fact, fluency may be measured by using various indexes: some of them may consider the percentage of stuttered syllables only, others (e.g., SSI-4) consider also physical concomitants and the duration of dysfluencies (perhaps resulting in greater levels of variability, especially among different raters) (see Tahmasebi et al., 2018). Finally, other scales mainly rely on a “subjective” evaluation of the participants (e.g., OASES; however, aspects related with the perceived quality of life, as well as affective/social and cognitive/behavioral characteristics of DS, are equally important to define the severity of the disturbance). A second aspect may be related to evidence that also the investigated protocols are heterogeneous in case of considering both brain targets and the characteristics of stimulation. In this context, the recent observations of Neef et al. (2021) suggest that, in stuttering, assisted (behavioral) fluency recovery that mainly supports neural compensation rather than the normalization of speech/motor circuits should be also taken in account to better evaluate the outcomes of neuromodulation effects on brain networks. In fact, neural circuits are continuously influenced by the actual state dependency of the brain (e.g., Silvanto and Pascual-Leone, 2008; Bergmann, 2018) that, as a consequence, may be a further element of variability to be taken into consideration in case of evaluating the neurophysiological effects of treatments.

However, all reviewed works, especially those showing that some positive effects arise also after a single stimulation session (and might endure at follow-up), deserve further consideration, investigation, and replication. As a matter of fact, current insights may be very useful not only to improve speech interventions in DS, but also to obtain a further and better understanding of neural dynamics involved in stuttering. This should be done because the use of neuromodulatory NIBS, in DS, is very recent: protocols need to be optimized to better understand their effects and interactions with brain functioning.

On one hand, findings suggest that increasing the neural activity of the left inferior frontal cortex may help in improving speech fluency (Chesters et al., 2017, 2018; Le Guilloux and Compper, 2018; see Figure 1). This is fully compatible with the previous evidence of wide structural and functional dysfunctions of this particular brain region (and related neural networks) in DS (e.g., Sommer et al., 2002; Watkins et al., 2008; Neef et al., 2016; Desai et al., 2017; Busan et al., 2019; see also Etchell et al., 2018, for a comprehensive review): the left inferior frontal cortex is classically thought to be involved in speech processing, strongly contributing to speech/motor plans that should be used to “feed” sensorimotor cortices (e.g., Neef et al., 2016). For example, as already suggested, this could be possible thanks to the FAT fascicle, connecting this region with the SMA “complex” (e.g., Catani et al., 2012; Dick et al., 2019; La Corte et al., 2021).

On the other hand, the inhibition of the homologous regions of the right hemisphere also resulted in improved levels of speech fluency, especially in case of considering reading tasks (Yada et al., 2019; Tezel-Bayraktaroglu et al., 2020; see also Figure 1). Conversely, the speech during conversational tasks resulted in lower fluency (Tezel-Bayraktaroglu et al., 2020). Evidence about the role of the right hemisphere in stuttering mainly suggests a compensatory role of these structures (e.g., Neumann et al., 2003; Preibisch et al., 2003). However, compensatory processes may result in “adaptive” or “maladaptive” mechanisms: as a consequence, discrete evidence of an abnormal (i.e., increased) structure and function of the right hemisphere, in DS, may also partially contribute to the maintenance of the disturbance and its pathophysiological mechanisms, speculatively due to excessive inhibitory mechanisms likely related to a conscious motor control (e.g., Neef et al., 2016, 2018). This may resemble the similar evidence highlighted in stroke-induced aphasia: the damaged speech/motor regions of the left hemisphere may be compensated by the intervention of the homologous regions of the right one (e.g., Hamilton et al., 2011; Balaev et al., 2016; Skipper-Kallal et al., 2017). Anyway, this hemispheric “disequilibrium” may also result in stronger inhibitory projections that arise from the “healthy” side of the brain toward the regions of the affected one (see Hamilton et al., 2011). For this reason, aphasia may usually benefit from TMS/tES inhibitory interventions on the brain regions of the (healthy) right hemisphere of patients, thus favoring the left hemispheric “re-activation” (e.g., Martin et al., 2004; Naeser et al., 2005a,b; Hamilton et al., 2010, 2011; Kang et al., 2011; Marangolo et al., 2013c). Nevertheless, the findings of Yada et al. (2019) and Tezel-Bayraktaroglu et al. (2020) suggest that the speech fluency of the reading and conversation tasks may be differently treated in DS, speculatively relying on different neural networks, especially in the right hemisphere: while the frontal right regions may be more related to compensation (and control) of the conversational and spontaneous speech, reading may also depend on “automatical” (or “rhythmic”) speech processing, in which “external” (i.e., sensorial) cues may involve additional neural circuits. Accordingly, Busan et al. (2020) showed that the signal-to-noise ratios of muscular activation and the intracortical inhibition of the right primary motor cortex are “improved” (especially) when people who stutter are facing the motor tasks “cued” by an external sensorial stimulation (i.e., an acoustic signal), thus suggesting a possible mechanism of efficacy for a series of fluency-inducing techniques, such as the “choral-speech” effect or the use of a metronome. However, more generally, this vision is also compatible with the evidence suggesting that the neural mechanisms that are useful for reading may be broader and widespread in various complex systems, which bilaterally involve fronto-temporoparietal networks, as well as precentral and postcentral areas (e.g., Roux et al., 2004; Morshed et al., 2020). In accordance with the finding, neuromodulation may differentially affect reading and conversation tasks: Crinion (2018) (referring to the evidence of stronger tDCS positive effects on reading-related dysfluencies, with respect to conversation, reported in Chesters et al., 2018) suggested the possibility that differences in task-related modulatory effects may exist, favoring “well-learned” and “well-practiced” mechanisms, such as those associated with reading, with respect to “less stable” activity that may be present in conversation tasks.

Another cortical region that seems to be strongly correlated to the positive effects of neuromodulation in DS is the SMA “complex” (Garnett et al., 2019a; Mejías and Prieto, 2019): the SMA is an associative motor region involved in the management of complex (internally driven) motor sequences, such as speech (e.g., Picard and Strick, 1996; Alario et al., 2006; Seitz et al., 2006). As already highlighted, the SMA is able to exchange information with different cortical structures, not only with the inferior frontal regions (by means of the FAT fascicle) (e.g., Dick et al., 2019) but also with subcortical structures such as the basal ganglia. In the latter case, it is part of an “*internal timing* (motor) *network*” that has been shown to be defective in DS, thus resulting in difficult preparation, initiation, and control of voluntary, “precise in time,” and “complex” motor sequences (see Alm, 2004; Etchell et al., 2014; compare with Chang et al., 2016; Busan, 2020). In contraposition, an “*external timing* (motor) *network*” also exists (mainly composed of structures such as the lateral premotor regions, the cerebellum, and the right inferior frontal regions) (see Alm, 2004; Etchell et al., 2014), which may be suggested to sustain the effectiveness of “fluency-inducing” conditions (especially in case of their characterization by the presence of a paced external rhythm) (compare with Alm, 2004; Etchell et al., 2014), also restoring a more “near-to-normal” and left-lateralized neural activity in people who stutter (e.g., Neumann et al., 2003; Giraud et al., 2008; Toyomura et al., 2011, 2015). As a consequence, the SMA may represent a critical neural “hub” in DS (see Busan, 2020), in which neuromodulation may allow to “restore” also associated cortical/subcortical networks that may similarly interfere with (motor) speech planning, initiation, and execution, in people who stutter.

Preliminary evidence suggests that the stimulation of the left temporal cortex (in case of its association with DAF) may also help in improving speech fluency, in DS (Moein et al., 2020)^1^, thus resulting in augmented task-related fluency effects (and, likely, in corresponding neural plasticity). Temporal cortex may also have a role in stuttering in a compatible manner: audio–motor interactions have been reported to be impaired in people who stutter (e.g., Beal et al., 2010, 2011; Cai et al., 2012, 2014a; Daliri and Max, 2015), whereas (especially) the right temporal cortex may be involved in ‘‘adaptive''/‘‘maladaptive” compensatory processes related to dysfluencies (e.g., Foundas et al., 2004; Jäncke et al., 2004; Beal et al., 2013; Busan et al., 2019). As a matter of fact, this region has been often reported as characterized by structural or functional abnormalities, in DS (see Etchell et al., 2018, for a general review). For example, stuttering may be characterized by locally increased (or lowered) gray matter volumes of the bilateral temporal cortices (e.g., Beal et al., 2007; Song et al., 2007; Chang et al., 2008). In this context, lower hemispheric asymmetries may be also evident (e.g., Foundas et al., 2001, 2004; Jäncke et al., 2004), and task-dependent functional abnormalities (i.e., lower or higher activity) are often reported (e.g., De Nil et al., 2008; Chang et al., 2009; Ingham et al., 2012; Lu et al., 2017). Moreover, this region is part of the speech motor network thanks to discrete connection fibers, such as the left arcuate fasciculus, that may result in lower white matter integrity in people who stutter (see Garnett et al., 2019b, for a recent review; see also Cai et al., 2014b; Cieslak et al., 2015; reports of an increase in white matter under the left temporal regions are also available, see Beal et al., 2007; Cai et al., 2014b). These defective patterns may easily result in altered or abnormal connectivity, for example, with the basal ganglia or SMA (e.g., Lu et al., 2010; Cieslak et al., 2015; Yang et al., 2016; see also Chang and Zhu, 2013), as well as with the inferior frontal regions (Chang et al., 2011), and sometimes also resulting in correlations with stuttering severity (e.g., Cai et al., 2014b). A neural model suggests that DS may appear from an excessive “overreliance” of the neural system on auditory feedbacks, with consequent delays in speech/motor activations: the lack of correct auditory feedbacks may lead to a restarting of the intended motor programs, thus resulting in dysfluencies (Civier et al., 2010).

However, current evidence more properly suggests that DS should be considered as a “dynamic” timing and motor control disorder, affecting broader neural networks in the brain and their communications (e.g., Ludlow and Loucks, 2003; Alm, 2004; Etchell et al., 2014). Dysfluencies may be the result of “poor” neural synchronization (or “delayed” neural activation) among different brain regions (see Salmelin et al., 2000; Etchell et al., 2014; Busan et al., 2019), altering the balance among excitatory and inhibitory (motor) signals (e.g., Busan et al., 2016, 2017, 2020).

In this context, the neural modeling of DS suggests that stuttering may be the result of impaired feedforward processing of speech/motor programs (e.g., Postma and Kolk, 1993; Howell, 2004; Max et al., 2004; Giraud et al., 2008; Civier et al., 2010, 2013; Packman, 2012; Chang and Guenther, 2020). For example, Giraud et al. (2008) propose the existence of a defective exchange of the information between the cortico-basal-thalamo-cortical circuits and the motor/speech regions of the left hemisphere: the homolog cortices of the right hemisphere may compensate for these defects, but likely resulting in a “delayed” neural activity, and thus in stuttering (see also Busan et al., 2019). Wu et al. (1995) proposed a comparable model also considering the cerebellum as a useful structure to correct motor timing deficits in DS and adding the limbic system as a possible emotional “modulator” (i.e., higher anxiety resulting in a worsened stuttering). Compatibly, it has been suggested that the anticipation of upcoming difficulties may lead to the setting of further higher neural thresholds for the subsequent release of the intended motor/speech plans (Brocklehurst et al., 2013; see also Smith and Weber, 2017) (for a model considering the effects of psychosocial and emotional factors, for example, the presence of a heightened “arousal,” in contributing to the appearance–and maintenance–of dysfluencies).

Successively, after the already cited model about “overreliance” on auditory feedbacks (Civier et al., 2010), Civier et al. (2013) considered the combined role of white matter impairments, premotor cortices, basal ganglia, and altered dopamine neurotransmission, in DS. Again, the authors concluded that the activity of the impaired neural networks may be “delayed” in stuttering, thus resulting in the abnormal timing and the exchange of neural information to facilitate dysfluencies. In this context, Chang and Guenther (2020) individuated three different “causal” alterations leading to compromised implementation of speech motor programs in people who stutter: impairments within the basal ganglia system, in the neural projections of the cortico-basal-thalamo-cortical networks, and in cortical processing.

Interestingly, a larger part of all these models may be compatible with the very recent suggestions of Alm (2021) and Turk et al. (2021). More specifically, they discuss evidence that DS may be the result of a metabolic disturbance (with a probable genetic basis—and in a mutual interaction with the dopaminergic brain systems, also useful for movement learning/automation), thus resulting in a deficit of energy supply to neurons, such as those that are part of the speech/motor networks (Alm, 2021). In this context, the importance of the role of astrocytes in modulating the dopaminergic networks involved in the implementation of normal/abnormal speech is also considered (Turk et al., 2021).

In summary, in all these models, the role of neural “hubs,” related to wider interconnected neural networks, such as the (left) inferior frontal cortex, the cortico-basal-thalamo-cortical system (including the SMA “complex”), or the temporal cortex (i.e., the regions that are fundamental for the correct motor/speech programming and execution), is evident. An effective communication among them is constantly needed through the discrete patterns of connections such as the FAT, the fascicles connecting anterior and posterior parts of the brain, and corpus callosum. In the end, bilateral inferior frontal cortices may be promising targets for a non-invasive neuromodulation in stuttering, with reversed effects in the two hemispheres. Attempts also suggest that acting on the temporal cortex or, especially, on the cortico-basal-thalamo-cortical networks of people who stutter, may be a promising approach to reduce speech dysfluencies. In the latter case, the SMA “complex” may be a more achievable cortical target for acting on “defective” brain dynamics, and likely for improving the functioning of these wider and complex neural networks.

## Neuromodulatory NIBS in DS: Future Perspectives

The evidence also suggests future perspectives of neuromodulatory NIBS in stuttering: attention should be paid to the implementation and the investigation of new and more focused protocols of interventions. In this context, it could be useful to increase an understanding of the neural effects of “pure” neuromodulatory NIBS trials (i.e., not in combination with “fluency-shaping” interventions) on the neural networks of the people who stutter (taking always into account the state dependency of the stimulated neural circuits) (e.g., Silvanto and Pascual-Leone, 2008; Bergmann, 2018).

Described interventions have been mainly proposed to adult male persons who stutter. However, there could be some differences in neurophysiologic profiles in case of considering women who stutter (e.g., Ingham et al., 2004; Busan et al., 2013; Chang and Zhu, 2013; Choo et al., 2016), or, perhaps, adolescents/children who stutter (see Etchell et al., 2018). In this very last case, unassisted (i.e., spontaneous) recovery may be a further confounding factor. For this reason, trials should be conducted in a part of the population unequivocally identified as future, persistent, adults who stutter (e.g., Walsh et al., 2018). Actually, the recovery from stuttering (in adults and children) may be associated with a further reorganization of brain circuits (e.g., Neef et al., 2021). For example, a reduced speaking-related functional connectivity between the speech/motor regions, such as the inferior frontal cortex and the SMA, may be evident in adults who recovered fluency (Kell et al., 2018), possibly resulting in a better functionality of the left inferior frontal region (see also Kell et al., 2009). In this context, a low involvement of circuits related to the SMA “complex” has been also demonstrated in the recovered children (e.g., Garnett et al., 2018). As a consequence, speculatively, also the possible presence of some differences in genetic and metabolic profiles among people who stutter -perhaps resulting in differences in neuroplasticity/neuromodulatory outcomes- should be further considered and investigated -compare with Paulus (2011b), Benito-Aragon et al. (2020), Chow et al. (2020), Alm (2021).

Thus, the most recent models of neural functioning in DS, as well as a better understanding of the altered brain functioning related to stuttering (e.g., involved brain rhythms and/or functional connectivity) (see Etchell et al., 2015; Jenson et al., 2020), should be used to implement more advanced and effective neuromodulation interventions in terms of both targeted brain regions and stimulation protocols (e.g., HD-tES for a higher focus of stimulation, tACS for exploiting discrete stimulation frequencies, or TMS H-coils to better stimulate deeper neural structures such as basal ganglia) (e.g., Popa et al., 2019). Finally, advancements in this field should be also useful to improve in an evidence-based manner, interventions currently available for DS (e.g., behavioral therapy, as well as the outcomes of other usable interventions, such as psychotherapy or pharmacotherapy), also considering their effects on involved neural circuits (e.g., the better comprehension of the mechanisms resulting in fluency facilitation—for instance, those related to “choral speech,” see Kalinowski and Saltuklaroglu, 2003—or, on the other hand, in the worsening of speech dysfluencies—such as those related to anxiety or emotional “arousal”; compare with Craig-McQuaide et al., 2014; Yang et al., 2017; Toyomura et al., 2018).

## Conclusions

In conclusion, neuromodulatory NIBS may be a promising and useful approach to “boost” more conventional interventions in stuttering, thus resulting in an improvement of speech fluency in a better way. At present, the stimulation of neural circuits comprising the inferior frontal cortex and the SMA “complex” may be the more effective approach. Secondarily, temporal cortex may be also considered for additional investigation regarding its potential to serve as a further neural target that is useful to improve DS (compare with Moein et al., 2020)^1^. However, considering that stuttering is a wider and dynamic motor disorder (Ludlow and Loucks, 2003), involving sensorimotor regions and neural networks useful to motor programming and control, research should focus on improving neuromodulatory interventions in terms of both protocols and the definition of neural targets. This should be done to assure new, tailored, and more successful interventions (in the shortest possible time, and in addition to the already available interventions), thus resulting in a higher improvement in the quality of life of people who stutter.

## Author Contributions

PB: manuscript conception, writing, editing, bibliographic search and elaboration, and conception and realization of figures and tables. BM: manuscript conception and editing, bibliographic search and elaboration, and conception and realization of figures and tables. FM: manuscript editing and conception and realization of figures and tables. GDB: manuscript conception and editing. GC: manuscript conception and editing and bibliographic search and elaboration. All authors contributed to the article and approved the submitted version.

## Conflict of Interest

The authors declare that the research was conducted in the absence of any commercial or financial relationships that could be construed as a potential conflict of interest. The handling editor is currently organizing a Research Topic with one of the authors PB.

## Publisher's Note

All claims expressed in this article are solely those of the authors and do not necessarily represent those of their affiliated organizations, or those of the publisher, the editors and the reviewers. Any product that may be evaluated in this article, or claim that may be made by its manufacturer, is not guaranteed or endorsed by the publisher.

## Footnotes

^1^Moein, N., Mohamadi, R., Rostami, R., Nitsche, M., Zomorrodi, R., Ostadi, A., et al. (under review). Investigation of the effect of Delayed Auditory Feedback and transcranial Direct Current Stimulation (DAF-tDCS) treatment for the enhancement of speech fluency in adults who stutter: a randomized controlled trial. *Res. Square*. doi:  10.21203/rs.3.rs-29391/v1
